# Efficient TALEN-mediated gene knockin at the bovine Y chromosome and generation of a sex-reversal bovine

**DOI:** 10.1007/s00018-021-03855-1

**Published:** 2021-05-28

**Authors:** Ming Wang, ZhaoLin Sun, Fangrong Ding, Haiping Wang, Ling Li, Xue Li, Xianjin Zheng, Ning Li, Yunping Dai, Changxin Wu

**Affiliations:** 1 College of Animal Science and Technology, China Agricultural University, No. 2 Yuanmingyuan Xilu, Beijing, 100193 China; 2 College of Biological Sciences, China Agricultural University, No. 2 Yuanmingyuan Xilu, Beijing, 100193 China; 3 Beijing Capital Agribusiness Future Biotechnology Co, 75 Bingjiaokou Hutong, Ltd, 100088 No China; 4 Cattle Breeding Research Institute of Beijing Shunxin Xinyuan Co, 3 Anping Street, LtdShunyi District, 101318 No China

**Keywords:** Bovine, Y chromosome, TALENs, Knockout, Knockin, *Sry* gene, Sex reversal

## Abstract

Functional elucidation of bovine Y-chromosome genes requires available genome editing technologies. Meanwhile, it has yet to be proven whether the bovine *Sry* gene is the main or single factor involved in the development of the male phenotype in bovine. Here, we efficiently knocked out four Y-linked genes (*Sry*, *ZFY*, *DDX3Y*, and *EIF2S3Y*) in bovine fetal fibroblasts (BFFs) with transcription activator-like effector nucleases (TALENs) individually. Furthermore, we used TALEN-mediated gene knockin at the *Sry* gene and generated a sex-reversal bovine by somatic cell nuclear transfer (SCNT). The resulting bovine had only one ovary and was sterile. We demonstrate, for the first time, that the *Sry* gene is an important sex-determining gene in bovine. Our method lays a solid foundation for detecting the biology of the bovine Y chromosome, as it may provide an alternative biological model system for the study of mammalian sex determination, and new methods for the practical application in agricultural, especially for sex predetermination.

**Supplementary Information:**

The online version contains supplementary material available at 10.1007/s00018-021-03855-1.

## Introduction

The Y chromosome has important specialized functions in male sex differentiation, spermatogenesis, fertility, and related human health issues and diseases [1]. Manipulating Y-chromosome genes is a direct way to investigate their functions. However, perhaps due to the unique structural features of the Y chromosome, to date research on the mutation of Y-linked genes has been scarce and has been performed only in mice and rabbits with insertional targeting vectors [2], transcription activator-like effector nucleases (TALENs) [3–5], and CRISPR/Cas9 [6–9]. Large animals, especially bovine, are important model and valuable agricultural animals, and the use of genetic modification to establish an alternative biological model for the study of mammalian sex determination or achieve more precise pre-determine the sex [10], is a popular research topic. Therefore, a comprehensive approach to study the function of Y-linked genes in large animals is needed to fully elucidate the biology of the Y chromosome and improve related agricultural applications. To the best of our knowledge, no living large animals with gene knockout or gene knockin on the Y chromosome have been reported. Although, editing of the Y-chromosome specific gene *Sry* in pig primary cells through nonhomologous end joining (NHEJ) and loxP knockin has been reported, no individual animals have been generated [11, 12]. Importantly, whether the *Sry* gene is an important gene for sex determination in bovine, as it is in mice or rabbits [2], is still unclear.

To establish the application of TALEN-mediated gene editing for the Y chromosome in bovine, the bovine *Sry* gene was chosen as the target gene as the sequence and the expression of the bovine *Sry* gene has been described [13], and the gene has been reported to be a single-copy gene on the Y chromosome [14]. Importantly, the *Sry* gene serves as a main genetic switch for male sex development in mice and human [1], and well-defined function in testicular determination in mice and rabbits [2], and the *Sry* gene is also expressed in the bovine sperm [15], therefore, this locus may be an ideal site for gene knockin to ensure the expression of gene elements of interest on the Y chromosome, and to establish simpler, better systems for sexing sperm, such as the transgene-based sexing system [16].

Here, we report, for the first time, the use of TALEN-mediated gene editing to efficiently knock out Y-linked genes in bovine fetal fibroblasts (BFFs). Notably, efficient knockin of the *Sry* gene with TALENs was accomplished with a 9-kb dsDNA donor pSRY-EGFP construct containing Thosea asigna virus 2A (T2A) [17] with incorporation of EGFP (T2A-EGFP) and the positive selection marker neomycin (NEO) flanked by short homologous arms. Interestingly, while we attempted to use sperm-specific expression of a “suicide” gene to specially ablate the Y-sperm and achieve sex-control, the other dsDNA donor pSRY-DTA containing the “suicide” gene diphtheria toxin fragment A (DTA) gene [18] and driven by the bovine sperm-specific Protamine 1 (bPrm1) promotor [19], was non-precise-homologous recombination (NHR) knockin behind the *Sry* gene stop codon. Using the NHR-positive gene-knockin cells as donor, we generated a viable sex-reversal cow by nuclear transfer. The cow had only one ovary and was sterile. The method lays a solid foundation for detecting the biology of the bovine Y chromosome, as it may provide an alternative biological model system for the study of mammalian sex determination and new methods for practical agricultural applications, especially sex predetermination.

## Results

### TALEN-mediated gene targeting in BFFs

**Fig. 1:**
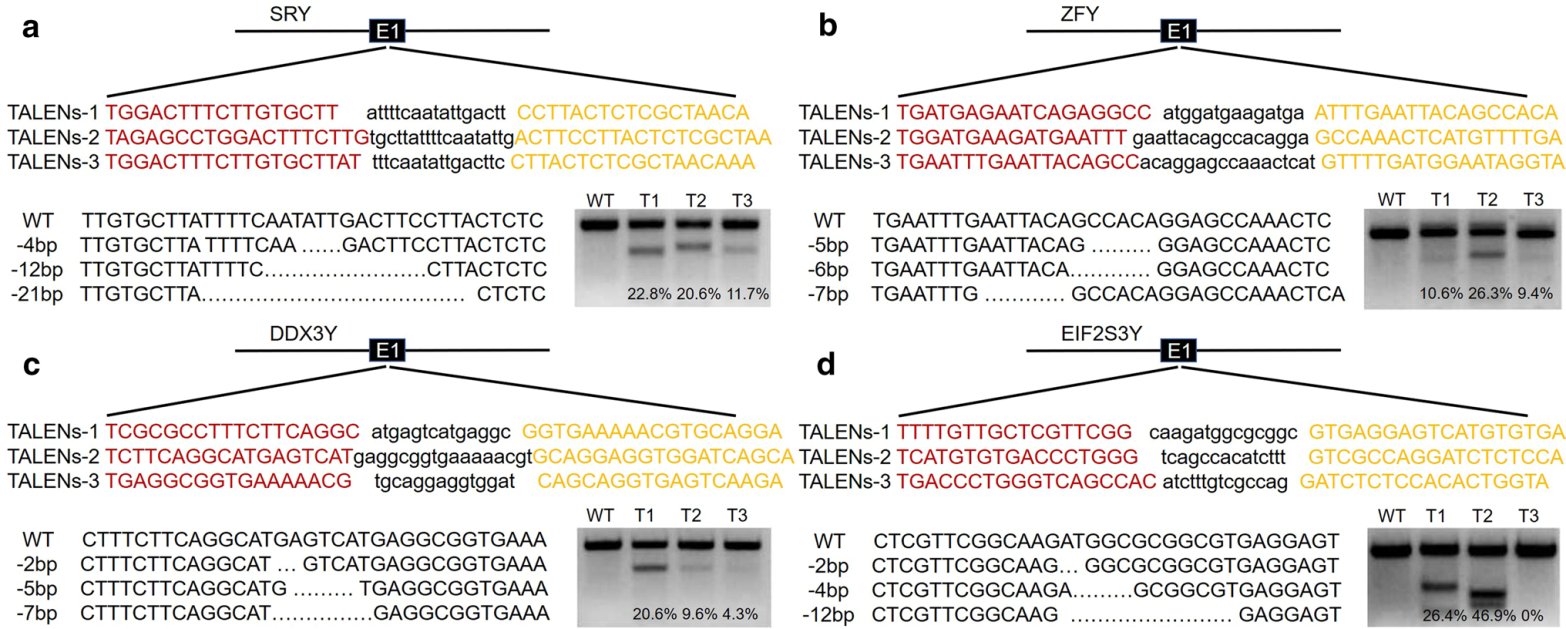
Design of TALENs for induction of DNA double-strand breaks (DSBs) in different bovine Y-chromosome genes. **a** Modification of the bovine Y-chromosome *Sry* gene by TALENs. The recognition sequences for the three TALEN pairs used in this study are highlighted: the left recognition sequence is highlighted in red, and the right recognition sequence is highlighted in yellow. Representative sequencing results of the TA clones revealing different indel mutations mediated by TALENs in the target site are shown at the lower left. Representative results of T7EI assays, which were used to detect the mutation efficiencies of different TALEN pairs, are shown at the lower right. The mutation frequencies (% indels) were calculated by measuring the band intensities. E1, Exon 1 of each gene (the TALEN-targeting locus); WT, wild-type control cells; T1, TALEN pair 1-transfected cells; T2, TALEN pair 2-transfected cells; T3, TALEN pair 3-transfected cells. **b** Modification of the bovine Y-chromosome *ZFY* gene by TALENs. **c** Modification of the bovine Y-chromosome *DDX3Y* gene by TALENs. **d** Modification of the bovine Y-chromosome *EIF2S3Y* gene by TALENs

First, we engineered three pairs TALENs directed against the bovine *Sry* gene instead of using a conventional gene-targeting strategy based on homologous recombination (Fig. 1a). We used a T7 endonuclease I (T7EI) to test the nuclease activity of these TALENs on the *Sry* gene in BFFs. TALEN pairs 1, 2, and 3 showed gene modification efficiencies of 22.8%, 20.6%, and 11.7%, respectively, and the modifications were subsequently verified by TA cloning and sequencing (Fig. 1a). Subsequently, we detected whether the same targeting strategies could be applied to other Y-linked genes and whether the efficiencies for the different genes were different. Zinc finger protein, Y-linked (*ZFY*), Asp–Glu–Ala–Asp box polypeptide 3, Y-linked (*DDX3Y*) and eukaryotic translation initiation factor 2, subunit 3, structural gene Y-linked (*EIF2S3Y*), which were single-copy genes in the male-specific region of the mammalian Y chromosome in bovine, were chosen [14]. The T7EI assay and sequencing analysis showed that the *ZFY*, *DDX3Y*, and *EIF2S3Y* genes could also be efficiently modified by TALENs (Fig. 1b–d). The characteristics of each TALEN pairs are provided in Table S1.

### TALEN-mediated gene knockin in BFFs

**Fig. 2:**
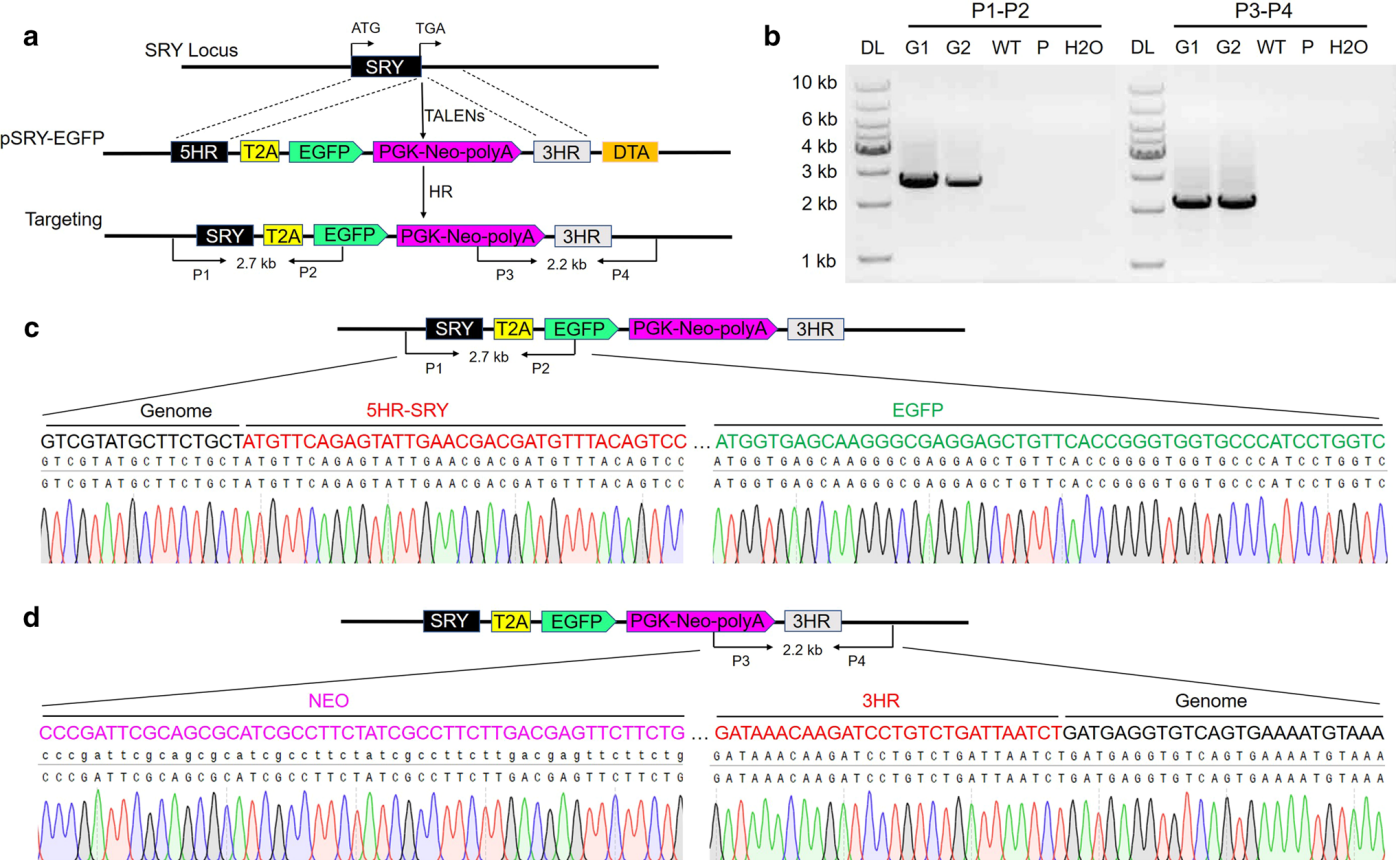
Production of the pSRY-EGFP-knockin cell clones by TALEN-mediated gene homologous recombination at the *Sry* locus. **a** Schematic overview of the strategy used to generate pSRY-EGFP-knockin cells in the bovine *Sry* gene. *Sry* TALEN pair 1 was used to introduce DSBs. The construct pSRY-EGFP was used as the homologous recombination donor. P1 and P2 are the primers outside the 5′ homologous arms; P3 and P4 are the primers outside the 3′ homologous arms. **b** PCR analysis of the pSRY-EGFP-knockin cells. The primer pairs are shown in Fig. 2a. Targeted inclusion of pSRY-EGFP resulted in amplification of an expected band of 2.7-kb from P1 and P2 and an expected band of 2.2-kb from P3 and P4. DL, 1-kb DNA ladder; G1-G2, the pSRY-EGFP knockin-positive cells; P, donor vector; WT, wild-type cells; H_2_O, negative control. **c** Sequencing confirmation of the 5′ junction after targeted integration of pSRY-EGFP cassettes into the bovine *Sry* gene. **d** Sequencing confirmation of the 3′ junction after targeted integration of pSRY-EGFP cassettes into the bovine *Sry* gene

**Fig. 3:**
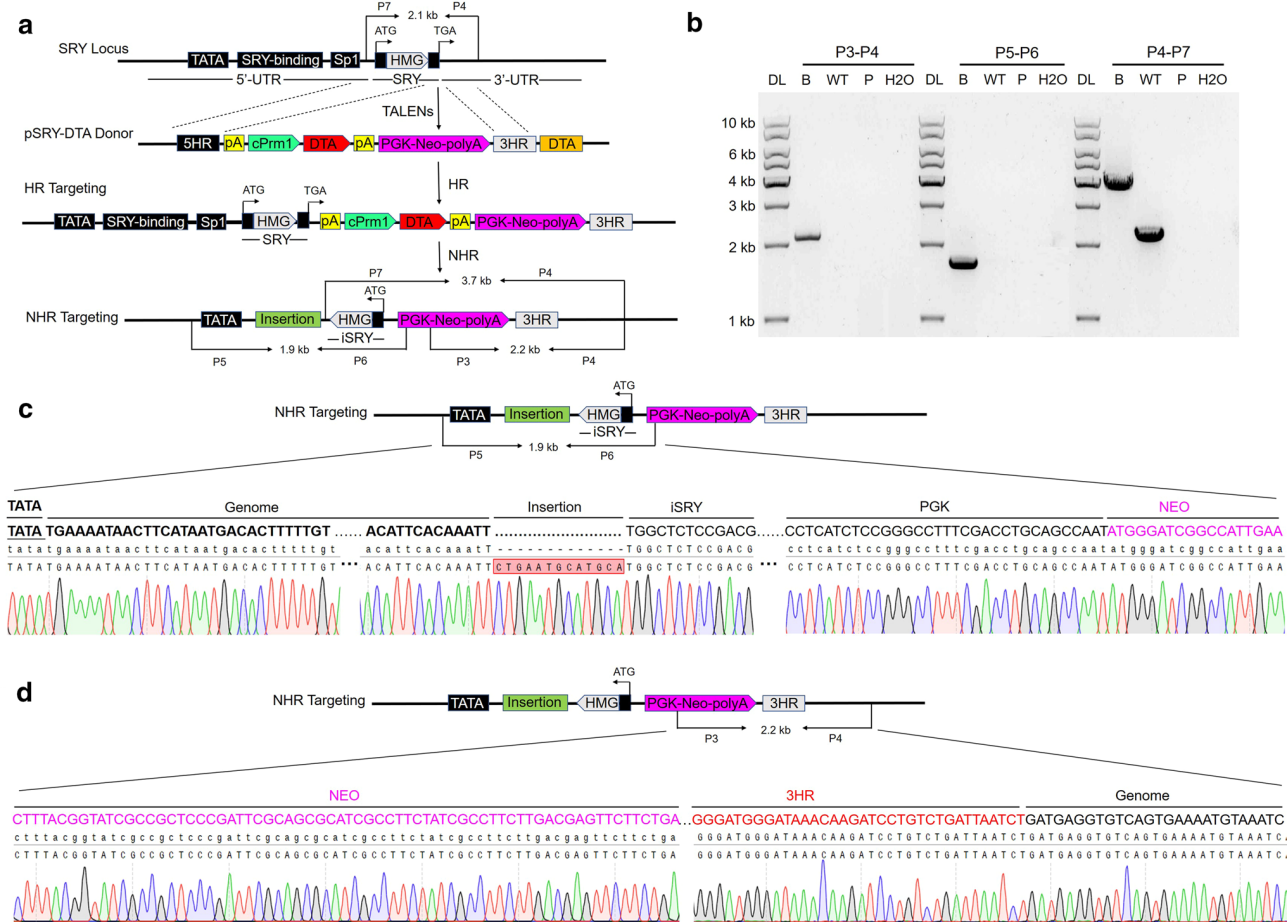
Production of the pSRY-DTA-knockin cell clones by TALEN-mediated gene nonhomologous recombination at the *Sry* locus. **a** Schematic overview of the strategy used to generate pSRY-DTA-knockin cells in the bovine *Sry* gene. *Sry* TALEN pair 1 was used to introduce DSBs. The construct pSRY-DTA was used as the homologous recombination donor. P5 and P6 are the primers outside the 5′ homologous arms; P3 and P4 are the primers outside the 3′ homologous arms. P4 and P7 are the primers outside the 5′ and 3′ homologous arms. **b** PCR analysis of the pSRY-DTA-knockin cells. The primer pairs are as shown in Fig. 3a. Targeted inclusion of pSRY-DTA resulted in amplification of an unexpected band of 1.9-kb from P5 and P6, an expected band of 2.2-kb from P3 and P4, and an unexpected band of 3.7-kb from P4 and P7. Using P4 and P7 also amplified a 2.1-kb band in WT cells. DL, 1-kb DNA ladder; B, the pSRY-DTA non-precise-homologous recombination (NHR) knockin-positive cells; P, donor vector; WT, wild-type cells; H_2_O, negative control. (c) Sequencing confirmation of the 5′ junction after targeted integration of pSRY-DTA cassettes into the bovine *Sry* gene. (d) Sequencing confirmation of the 3′ junction after targeted integration of pSRY-DTA cassettes into the bovine *Sry* gene

To further establish the applicability of TALEN-mediated editing for the Y chromosome in bovine, we first produced a TALEN-mediated GFP-knockin allele at the bovine *Sry* locus. The gene-targeting vector pSRY-EGFP was constructed as shown in Fig. 2a. We introduced *Sry* TALEN pair 1, along with a 9 kb dsDNA donor construct pSRY-EGFP that contained the self-cleaving peptide the 2A regions of Thosea asigna virus (T2A) incorporated EGFP (T2A-EGFP) and a positive selection marker Neomycin (NEO) flanked by short homologous arms (843 bp and 910 bp for 5′ and 3′ arms, respectively) and a negative selection marker diphtheria toxin A (DTA), into the male bovine primary fibroblasts (mBFF1004, XY) (Fig. 2a). After transfection, mBFF1004 cells were selected with G418. As shown in Table S2, we screened 12 single-cell clones and obtained 2 correctly targeted clones by polymerase chain reaction (PCR) genotyping. The primers P1 and P2 amplified the expected 2.7-kb band, and the primers P3 and P4 amplified the expected 2.2-kb band (Fig. 2b). The PCR products were also identified by sequencing (Fig. 2c, d). The successful GFP-knockin at the *Sry* locus prompted us to test whether other unique gene elements could be knocked into the Y chromosome-linked gene locus. The donor vector pSRY-DTA, in which the DTA gene was driven by the bovine sperm-specific Protamine 1 (Prm1) promotor, was constructed and was determined to knockin behind the *Sry* gene stop codon (Fig. 3a). We introduced *Sry* TALEN pair 1, along with the donor construct that contained the element of interest and a NEO selection marker flanked by short homologous arms (843 bp and 910 bp for 5′and 3′arms, respectively), into mBFF1004 cells (Fig. 3a). After transfection, the mBFF1004 cells were selected with G418. As shown in Table S3, we screened 39 single-cell clones and obtained 1 NHR-targeted clone by PCR genotyping. The P3/P4 primer set amplified the expected 2.2-kb band, while the P5/P6 and P4/P7 primers sets amplified the unexpectedly truncated 1.9-kb band and 3.7-kb band, respectively (Fig. 3b). TA cloning and sequencing further confirmed the above results (Fig. 3c, d). Although the sex-control vector did not work, the NHR-targeted clone exhibited the editing of the *Sry*. The editing included deletion of two important fragments (the 179 bp 5′ untranslated region (5′-UTR) contained the SRY-binding and Spl-binding motifs and the 302 bp opening reading frame (ORF)), inversion of one important fragment (the 91 bp 5′-UTR and 388 bp ORF containing the HOM box), and a 13 bp extra base insertion (Fig. S1, S2). As the NHR-targeted clone exhibited partial deletion and universal *Sry*, it would help us to understand the function of the *Sry* gene in bovine. Therefore, that cell clone was used to perform nuclear transfer. As shown in Table 1, we produced a total of 1578 reconstructed embryos from clone 35#, 25.8% of which successfully entered the blastocyst stage.

**Table 1:** Summary of the nuclear transfer results

No. of cell clone	Oocytes	Reconstruct embryos	Blastocysts	Blastocysts %	Recipients	Pregnancy at day 60	Live-born calves
#35	2910	1578	407	25.8%	52	18	1

### Generation of a genetically engineered bovine via somatic cell nuclear transfer (SCNT)

**Fig. 4:**
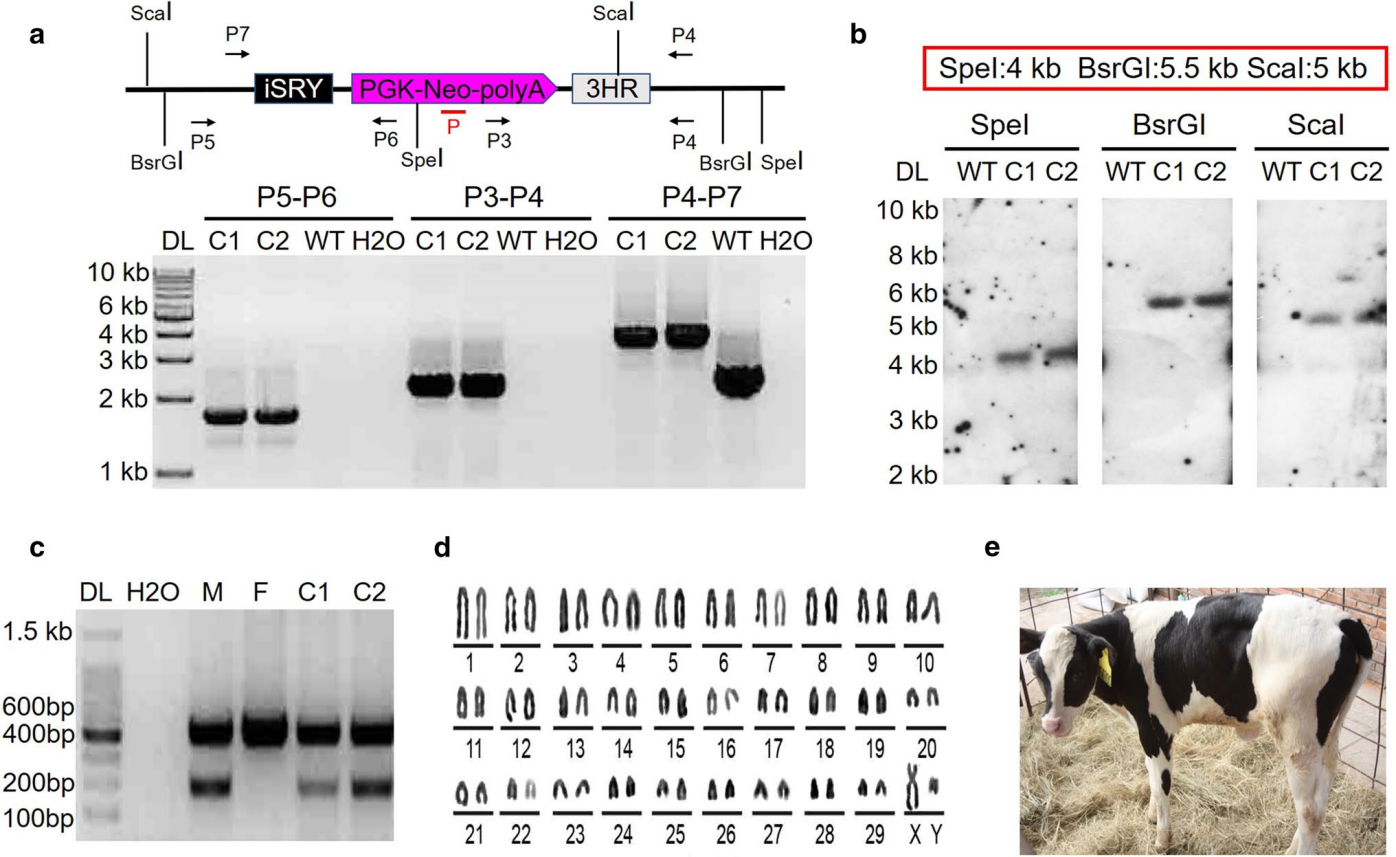
Generation of sex-reversal bovine by TALEN-mediated gene homologous recombination at the *Sry* locus. **a** Schematic of the analysis of the pSRY-DTA-knockin bovine. The primers P5 and P6 amplified a 1.9-kb product, the primers P3 and P4 amplified a 2.2-kb product, and the primers P4 and P7 amplified a 3.7-kb product to confirm positive pSRY-DTA-knockin cloned bovine. DL, 1-kb DNA ladder; C1 and C2, the pSRY-DTA NHR knockin-positive heifers (C1 represents #160,517, and C2 represents #160,705); WT, wild-type bull; H_2_O, negative control. The red line represents the DNA probe used for Southern blot analysis. The restriction endonucleases SpeI, BsrGI, and ScaI were used for Southern blot analysis. **b** Southern blot analysis of the gene-knockin bovine. The red rectangular box represents the positive band size obtained using different enzymes. Upon digestion with SpeI, a band of 4.0-kb resulting from targeted inclusion of pSRY-DTA was detected. Using the BsrGI, the expected fragment size was 5.5-kb; using the ScaI, the expected fragment size was 5.0-kb. C1 and C2, the pSRY-DTA NHR knockin-positive heifers (C1 represents #160,517, and C2 represents #160,705); WT, wild-type bull. **c** Sex determination by PCR method. The couple primers were used to amplify the bovine genomic DNA. For bulls, two fragments of 300-bp and 538-bp were amplified from the Y-specific primer and a bovine-specific primer, while for heifers, a clear single band of 538-bp was amplified. DL, 100-bp DNA ladder; C1, sex-reversal heifer #160,517; C2, sex-reversal heifer #160,705; M, wild-type bull; F, wild-type heifer; H_2_O, negative control. **d** Representative image of the XY karyotype of sex-reversal heifer C2 (#160,705). **e** Image of sex-reversal heifer C2 (#160,705)

In total, we transferred 100 transgenic cloned blastocysts into 52 recipients, and a total of two cloned calves were born. We numbered them by date of birth #160,517, #160,705. However, #160,517 died soon after birth, only #160,705 remained alive. The calves were positive for gene knockin, as confirmed by PCR genotyping (Fig. 4a) and Southern blot analysis (Fig. 4b). Sex determination by PCR confirmed that the two gene-knockin calves were male (Fig. 4c). Karyotyping of the sex-reversal heifer’s cells (#160,705) revealed 60 chromosomes, including XY (Fig. 4d). Meanwhile, as shown in Table S4, the expected allele sizes for the eleven bovine-specific microsatellite loci from the sex-reversal heifer #160,705 were consistent with those of the donor cell line (mBFF1004, XY), confirming that the sex-reversal heifer and the donor cell line (mBFF1004, XY) were from the same genetic source. The sex-reversal heifer #160,705 was healthy (Fig. 4e). To test for the potential nonspecific mutations induced by the introduction of the TALENs, 10 potential off-target loci of TALEN pair1 were predicted (Table S5). Sanger sequencing and T7EI analysis were used to determine the off-target effects. The results revealed that no off-target effects occurred in the sex-reversal heifer #160,705 (Fig. S3).

### Phenotype identification of the sex-reversal bovine

**Fig. 5:**
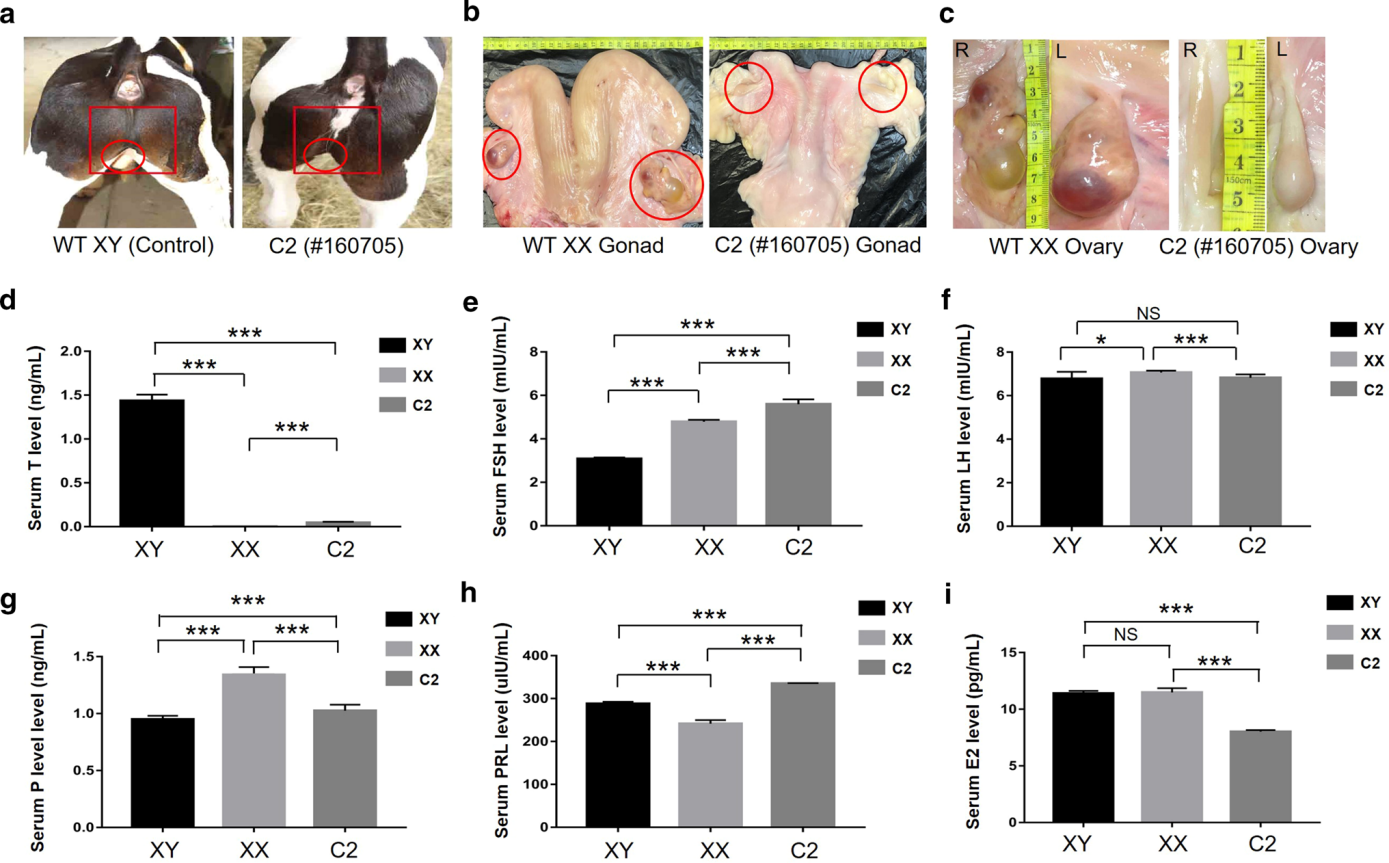
The ovary and sex hormone detection of the sex-reversal bovine. **a** Image of the sex-reversal heifer. **b** The reproductive organs of the sex-reversal and the WT, XX cow. **c** The ovaries of the sex-reversal and the WT, XX cow. **d–i** Sex hormones were determined using ELISA. XY, the wild-type bulls; XX, the wild-type heifers; 160,705, the sex-reversal heifer. The results are presented as the mean ± SEM. *p < 0.05; **p < 0.01; ***p < 0.001; NS, not significant

When the heifer #160,705 was 1 year old, we examined the external genitalia of the sex-reversal heifer. As shown in Fig. 5a, the heifer had female external genitalia. When #160,705 was 4 years old, we examined its internal genitalia. Compared with wild-type (WT) cow, the XX cow #160,629, #160,705 had significantly smaller genitalia, with thinner uterine horns and underdeveloped ovaries (Fig. 5b). The left ovary was solid with few follicles, while the right ovary was fasciculate and had few ovarian structural features (Fig. 5c). No intact follicles were observed by hematoxylin and eosin (H&E) staining in the #160,705 ovary, further verifying the developmental disorder of the ovarian interstitial cells (Fig. S4). The absence of follicles indicated ovulatory dysfunction in sex-reversal cow #160,705.

### Fertility of the sex-reversal bovine

To test the fertility of the sex-reversal heifer #160,705, we mated it when it was sexually mature with WT bull in which fertility had been previously confirmed. However, we did not detect pregnancy in #160,705, meanwhile it had no apparent oestrus or copulatory behavior like that of normal females. The animal did not become pregnant even after injection with the ovulation-stimulating hormones follicle-stimulating hormone (FSH) and luteinizing hormone (LH) before mating. In addition, we tried to obtain fertilized eggs by in vitro fertilization (IVF) using the ovum pick-up (OPU) method; unfortunately, oocytes could not be obtained. We concluded that the infertility of the sex-reversal cow was due to the abnormal development of the ovaries. This finding was consistent with those of previous studies, suggesting that infertility was a common symptom of male-to-female sex-reversal syndrome [20].

### Hormone levels in the sex-reversal bovine

Sex hormones play a crucial roles in follicle formation, embryo implantation and pregnancy. To examine whether the sex-reversal heifer had endocrine features characteristic of females, the levels of six sex hormones were tested using ELISA. The testosterone (T) levels in #160,705 were significantly lower than those in the WT XY controls and were in the range of those in the WT XX controls (Fig. 5d). Reduced levels of estradiol (E2), progesterone (P), and luteinizing hormone (LH), which could stimulate the development and maintenance of female reproductive tissues, were detected in the #160,705 (Fig. 5g–i). However, elevated levels of prolactin (PRL) and follicle-stimulating hormone (FSH) were observed in #160,705 (Fig. 5e, f). These results suggested that the ovarian developmental abnormalities might have resulted in aberrant levels of sex hormones, leading to sterility in sex-reversal cow #160,705.

## Discussion

In this study, we found that Y-linked genes can be efficiently targeted by TALEN-mediated gene-editing strategies in BFFs. In addition, we successfully established a TALEN-mediated method for efficient long dsDNA donor knockin in the *Sry* locus of the bovine Y chromosome and generated a viable bovine with sex reversal. For the first time, we used TALENs to edit Y chromosome-linked genes such as *Sry*, *ZFY*, *DDX3Y*, and *EIF2S3Y* in bovine, and the highest efficiency for different TALEN pairs were 22.8%, 26.3%, 20.6%, and 46.9%, respectively. The editing efficiency for the *Sry* gene was similar to that in mouse embryonic stem (mESCs) cells [3]. This finding proves that TALENs can be applied for bovine Y chromosome-linked genes editing. This method is expected to aid in elucidation of the biological function of the Y chromosome. Recently CRISPR/Cas9 technology has been widely used to generate gene-editing animals and destroys the entire chromosome in mice [6, 7, 21]. Although the use of TALENs is feasible in bovine, it is far inferior to the use of Cas9, and it is difficult to perform multigene manipulation and targeted deletion on the Y chromosome with TALENs. Therefore, CRISPR/Cas9 technology could be used to further improve livestock Y-chromosome research in the future. Gene knockin on the Y chromosome has a wide range of applications. Y-chromosome gene knockin was first performed in mESCs by TALENs and traditional gene-targeting vectors [3]. However, the gene-knockin efficiency was low (1%) in the *Sry* gene. We used the same method to obtain BFF clones with knockin in the *Sry* gene and accomplished knocking with greater efficiency than the previous study (16.7%). The differences in efficiency may have been due to the different structures of the chromatin in which the *Sry* genes of mice and bovine are located. Recently, a modified loxP (mloxP) site was efficiently integrated into *Sry* loci in pig fetal fibroblasts with TALENs and ssDNA [12]. However, since ssDNA is very short (tens of nucleotides), it’s difficult to detect the random integration of ssDNA into genome; deep sequencing might be necessary to evaluate the random integration of ssDNA. We are currently trying to obtain a longer donor template (9 kb) to mediate the site-specific insertion of long fragments, which will address future needs. We have also attempted to establish a sex-control technology by knocking in a “suicide” element including an SV40 polyA-bPrm1-DTA-bGH polyA construct into the *Sry* gene, in which the DTA gene was drived by the bovine sperm-specific Protamine 1 (bPrm1) promotor behind the Sry gene stop codon. We speculated that the sperm-specific expression of the DTA gene in the Y chromosome may specially ablate the Y-sperm, to achieve sex-control. In the process of cell screening, we found that the positive HR-targeted clone containing DTA integration could not be obtained, only one NHR-targeted clone was obtained. This outcome may have been caused by leaky expression of small amounts of sperm-specific DTA in BFFs, consistent with the findings of previous reports [22]. As the NHR-targeted clone exhibited partial deletion and universal *Sry*, we thought this would help us to understand the function of the *Sry* gene in bovine. Therefore, we continued cloning and verify whether the *Sry* gene is the sex-determining gene in bovine from the individual level of animals. Using the NHR-targeted clone, we generated a bovine with *Sry* gene editing that displayed sex reversal, consistent with the outcomes of mouse and rabbit research. Our bovine with sex reversal had only one ovary and was infertile, which is consistent with finding in mice. Our findings suggest that the *Sry* gene is the main factor in the development of the male phenotype in bovine. Although we did not detect off-target mutations in any of the potential off-target sites in our sex-reversal bovine using DNA sequencing and T7E1 assay, however, off-target effects could not be ruled out. As a recent study on genetic analyses of a TALEN-mediated gene-knockin hornless bull found that the entire plasmid was unintended inserted into the animals, but this result had not been found in their previous studies because the reads of the plasmids were discarded as unmapped, resulting in false negative results [23]. Therefore, the whole-genome sequencing technology can be used for off-target detection, especially HDR plasmid was involved, or the new low off-target Cas9 system can be used in later studies [24–26].

In summary, we established gene knockout and knockin techniques for the bovine Y chromosome. Furthermore, we found that the function of the *Sry* gene is important in male sex determination in bovine. Our results suggest that TALEN-mediated gene editing will enable the study of Y chromosome biology via genetic manipulation in other agricultural animals and may improve the application of biomedical and agricultural approaches in the future.

## Materials and methods

### Animals

Ovaries from slaughtered mature cows were collected from local abattoir in Beijing, China. Mature control and SCNT recipient cows were obtained from Dairy Cow Center of Beijing. The experimental bovines were housed in stalls with free access to food and water. The bull and the cow of high genetic merit were mated to produce an elite male fetus that was recovered at day 60. The male bovine fibroblast cell line was isolated from this fetus via disaggregation of all tissue excluding the viscera and limbs, and cultured in Dulbecco’s Modified Eagle’s Medium (DMEM; Gibco, Grand Island, New York, USA) supplemented with 10% fetal bovine serum (FBS; Gibco, Grand Island, New York, USA) at 37.5 °C in an atmosphere of 5% CO_2_ and humidified air. The procedures were performed in strict accordance with the Guide for the Care and Use of Laboratory Animals. All the animal work in this study was approved by the Institutional Animal Care and Use Committee of the China Agricultural University under approval number SKLAB-2014–07-05. We performed all surgeries under sodium pentobarbital anesthesia and all attempts were made to minimize animal suffering.

### Vector constructs

The pPGKneoDTA vector was used as the backbone to construct the *Sry* targeting vector. A 843 bp 5’ homologous arm and a 910 bp 3’ homologous arm were amplified by PCR from the genome of male BFFs, and cloned into the pPGKneoDTA vector. The T2A-EGFP element and the SV40 polyA-cPrm1-DTA-bGH polyA were synthesized by Sangon Biotech. Conventional molecular cloning and Golden Gate assembly were used to construct the pSRY-EGFP and pSRY-DTA vectors. The linearized donor pSRY-EGFP and pSRY-DTA were obtained by AhdI digestion. TALEN expression constructs were assembled by Viewsolid Biotech Company (Beijing, China).

### Transfection of the primary BFFs

The male primary BFFs were established as described in the “Animals” section. Then, 8 µg TALEN pair 1 and the 4 µg linearized pSRY-EGFP or pSRY-DTA donor were nucleofected into 1 × 10^6^ BFFs using Amaxa Nucleofector reagent (Lonza Group AG, Basel, Switzerland) according to the manufacturer’s guidelines. The program T-016 was selected. After 48 h, the transfected cells were transferred to 10-cm plates with 10% FBS containing G418 (500 mg/ml) at a density of approximately 1 × 10^5^ cells/plate. Individual cell clones were isolated 7–14 days after G418 (500 mg/ml) selection, expanded, sequenced and cryopreserved after a total of 12–14 days in culture.

### T7EI assay

The editing activity of each TALEN was assayed using T7EI (New England Biolabs, Ipswich, MA, USA) as described previously [27]. Briefly, genomic DNA from TALEN-treated cells was extracted using a DNeasy Blood and Tissue kit (Qiagen, Hilden, Germany). PCR amplicons including nuclease target sites were generated using the following primer pairs: Sry-F/Sry-R for the *Sry* locus; ZFY-F/ZFY-R for the *ZFY* locus; DDX3Y-F/DDX3Y-R for the *DDX3Y* locus; EIF2S3Y-F/EIF2S3Y-R for the *EIF2S3Y* locus. All the primers are listed in Table S6. PCR products were then denatured, rehybridized, digested with the T7EI, and analyzed by agarose gel electrophoresis. The mutation frequencies (% indels) were calculated by measuring the band intensities. The bands were quantified based on the relative band intensities using ImageJ software.

### Identification of the positive cell clones by PCR

To identify the positive cell clones, genomic DNA was extracted from a single cell clone using a DNeasy Blood and Tissue kit (Qiagen, Hilden, Germany). To confirm the successfully edited clones among the pSRY-EGFP-targeted cells, two pairs of primers targeting a sequence between the donor and outside the 5’ or 3’ homologous arm, respectively, were used: the P1/P2 primer pair was used for the 5’ arm and produced a 2.7-kb amplicon, and the P3/P4 primer pair was used for the 3’ arm and produced a 2.2-kb amplicon. To confirm the successfully edited clones among the pSRY-DTA-targeted cells, another two pairs of primers between the donor and outside the 5’ or 3’ homologous arm were used: the P3/P4 primer pair was used for the 3’ arm and produced a 2.2-kb amplicon, and the primer pair P5/P6 was used for the 5’ arm and produced a 1.9-kb amplicon. A primer pair targeting a sequence outside the 5’ and 3’ homologous arms, P4/P7, which produced a 3.7-kb amplicon, was also used to confirm the successfully edited cell clones. PCR was performed for 35 cycles at 94 °C for 30 s, 60 °C for 30 s, and 72 °C for 2–3 min with a hold at 72 °C for 10 min. The PCR products were sequenced by TA-Clone. All the primers are listed in Table S6.

### SCNT

SCNT was performed as described previously [28]. Briefly, transgenic cells were transferred into enucleated oocytes for the production of reconstructed embryos in vitro and then fused using the ECM® 2001 Electro Cell Manipulation System (BTX, San Diego, CA, USA). The reconstructed embryos were activated with 10 mg/ml cycloheximide and 2.5 mg/ml cytochalasin-D in CR1aa medium. Day-7 blastocysts were collected for future transplantation. A total of 100 blastocysts were transferred into 52 recipient cows. For each recipient, we transferred 1–2 transgenic cloned blastocysts. Pregnancy was detected by ultrasonography at 60 days and 180 days post-transfer.

### Identification of sex-reversal bovine

To identify the sex-reversal bovine, genomic DNA was obtained from the ear tissue of potential sex-reversal heifer and WT bull through phenol/chloroform extraction. For PCR analysis, three pairs of primers P3/P4, P5/P6, and P4/P7, were used to confirm successful gene editing in the bovine; these primer sets produced 2.2-kb, 1.9-kb, and 3.7-kb amplicons, respectively. PCR was performed for 35 cycles at 94 °C for 30 s, 60 °C for 30 s, and 72 °C for 2–3 min with a hold at 72 °C for 10 min. For Southern blot analysis, the genomic DNA (10 µg) was digested with the restriction enzymes SpeI, BsrGI, and ScaI, respectively, overnight. A digoxigenin-labeled probe that targeted the NEO gene was amplified using the PCR DIG Probe Synthesis Kit (Roche, Mannheim, Germany) with the primer pair neo-F/neo-R (Table S6). After 0.7% agarose gel electrophoresis for 6–8 h, the DNA was transferred to a nitrocellulose filter for blotting. The nitrocellulose membrane (Roche, Mannheim, Germany) was hybridized with a probe for 16 h and incubated with antibody for 0.5 h. The expected sizes of the positive bands using SpeI, BsrGI, and ScaI were approximately 4.0 kb, 5.5 kb, and 5.0 kb, respectively.

### Sex determination by PCR method

Sex determination was performed as previously described [29]. Briefly, multiplex PCR with two sets of PCR primers, Y-chromosome specific primers (BY-F/R), and bovine specific primers (BSP-F/R), was used to test the samples. The amplification reactions were conducted in a total volume of 50 ul using Q5 DNA polymerase (New England Biolabs, Ipswich, MA, USA). The amplification was carried out in a DNA thermal cycler with an initial denaturation at 98 °C for 5 min and then 45 cycles at 98 °C for 20 s, 52 °C for 30 s, and 72 °C for 30 s followed by final extension at 72 °C for 10 min. 10 ml of each PCR product was electrophoresed on a 2% agarose gel and stained with ethidium bromide.

### Karyotype analysis

Bovine fibroblasts were used for karyotyping. The fibroblasts were derived from bovine tails that were cut into small pieces and cultured for 7 days. Then, the fibroblasts were incubated with 200 ng/ml demecolcine (Sigma) for 1 h, resuspended in 0.075 M KCl at 37 °C for 10–30 min, and fixed with Carnoy’s fixative (25% acetic acid in methanol) for 30 min. The cells were plated on precleaned slides, and karyotype analysis was performed using VideoTest-Karyo3.1.

### Microsatellite analysis

Microsatellite analysis was performed on the sex-reversal heifer cells and the gene knockin-positive cell clone #35 based on the protocol for StockMarks® Horse, Cattle, and Dog Genotyping Kits (Applied Biosystems, Foster City, CA, USA). Eleven microsatellite loci located on different bovine chromosomes were first visualized with 3% agarose gel electrophoresis and further confirmed by capillary gel electrophoresis with fluorescently labeled amplimers and laser scanning using an ABI PRISM® 3100 Genetic Analyzer. The amplification results were determined using the Genotyper software plots of GeneScan software. Allele sizes were assigned using Genemapper version 4.0 (Applied Biosystems, Foster City, CA, USA).

### Prediction of off-target sites

The genome sequence (species name: Cow, Latin name: *Bos taurus*, reference genome version: Ruminant Reference Genome, 4.6.1/bos Tau7) was divided into a small units of 10 kb. The left and right sequences of *Sry* TALEN pair 1 were input into the Cutadapt linked Adapter program (https://cutadapt.readthedocs.io/en/stable/guide.html#linked-adapters) with full length matching, no indels allowed, and a maximum base mismatch of 5. Linked Adapter cyclically searched within each segmented small unit; that was, after a successful match, it searched again from the position after the match until another match could not be found. In the matching process, only results with spacing lengths greater than 10 and less than 30 were retained.

### Hematoxylin and Eosin (H&E) staining

H&E staining was performed as previously described [30]. Briefly, the tissues were fixed with 4% paraformaldehyde for 48 h, embedded in paraffin wax, sectioned onto slides, stained with H&E and then analyzed by microscopy (Nikon TS100).

### Sex hormone assay

When the sex-reversal heifer #160,705 was 1 year old, the sex hormone assay was conducted. Sexually mature and in estrus WT-XX heifers (n = 3) and WT-XY bulls (n = 3), which were of the same age as the experimental heifer, were chosen as control. And the WT-XX heifers (n = 3) were without pregnancy. Serum was obtained by precipitation and centrifugation. Sex hormones, including Testosterone (T), Estradiol (E2), Follicle-stimulating hormone (FSH), Luteinizing hormone (LH), Progesterone (P), and Prolactin (PRL), were measured using an ELISA Kit (IBL, Germany). All experiments were repeated three times. The data were expressed as the mean ± SEM.

### Statistical analysis

Statistical analysis of hormonal data was performed using GraphPad Prism version 5.0 for Windows. One-way ANOVA followed by Tukey’s multiple comparison test was used for comparisons between groups with normal distributions. Differences between experimental groups were regarded as significant when P ≤ 0.05.

## Supplementary Information

Below is the link to the electronic supplementary material.

Supplementary file1 (DOCX 4469 kb)

## Data Availability

All data generated or analyzed during this study are included in this published article and its supplementary information files.

## Abbreviations

TALENs: Transcription activator-like effector nuclease
BFF: Bovine fetal fibroblast
SRY: Sex determining region Y
ZFY: Zinc finger protein, Y-linked
DDX3Y: Asp-Glu-Ala-Asp box polypeptide 3, Y-linked
EIF2S3Y: Eukaryotic translation initiation factor 2, subunit 3, structural gene Y-linked
SCNT: Somatic cell nuclear transfer
WT: Wild type
