# Supplementary material for: Efficient TALEN-mediated gene knockin at the bovine Y chromosome and generation of a sex-reversal bovine
Source: Cell Mol Life Sci. 2021 May 28;78(13):5415–25. doi: 10.1007/s00018-021-03855-1 (PMC8257526; doi:10.1007/s00018-021-03855-1)
Supplement: Supplementary file 1 — Supplementary file1 (DOCX 4469 kb) [file 18_2021_3855_MOESM1_ESM.docx]

**SUPPLEMENTARY INFORMATION**

**Efficient TALEN-mediated gene knock-in at the bovine Y chromosome and generation of a sex-reversal bovine**

Ming Wang^1,2^, ZhaoLin Sun^1,2,3*^, Fangrong Ding^2^, Haiping Wang^2^, Ling Li^2^, Xue Li^2^, Xianjin Zheng^4^, Ning Li^2*^, Yunping Dai^2^^*^ ,Changxin Wu^1^

1. College of Animal Science and Technology, China Agricultural University, No.2 Yuanmingyuan Xilu, Beijing 100193, China.
2. College of Biological Sciences, China Agricultural University, No.2 Yuanmingyuan Xilu, Beijing 100193, China.
3. Beijing Capital Agribusiness Future Biotechnology Co., Ltd, No.75 Bingjiaokou Hutong, Beijing 100088, China.
4. Cattle Breeding Research Institute of Beijing Shunxin Xinyuan Co., Ltd, No.3 Anping Street, Shunyi District, Beijing 101318, China.

*. Correspondence and requests for materials should be addressed to Z. S. (email: [sunzhaolin@bjsnzz.com](mailto:sunzhaolin@bjsnzz.com)), N. L. (email: ninglcau@cau.edu.cn) or Y.D. (email:[daiyunping@sina.com](mailto:daiyunping@sina.com))

**
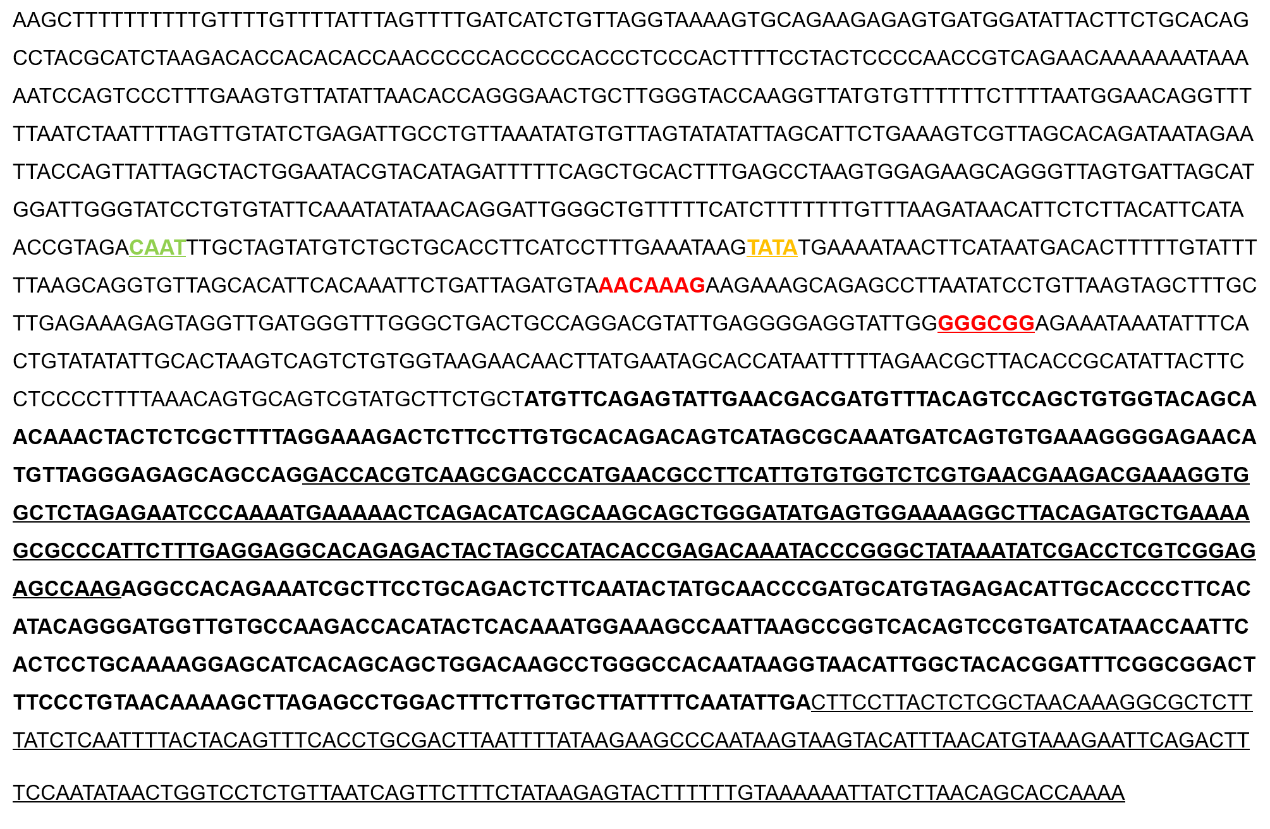
**

**Fig.S1** **Nucleotide sequence of the bovine *Sry* 1.8-kb genomic fragment**

This fragment included 911, 687, and 202-bp in its 5′ untranslated region (5′-UTR), Opening reading frame (ORF), and 3′ untranslated region (3′-UTR), respectively. The ORF is indicated in bold. The HMG box is underlined and bolded. In the 911-bp 5′-UTR, the TATA motifs is underlined and colored yellow, the CAAT box is underlined and colored green, the SRY-binding motif is colored in red, and the Spl-binding motif is underlined and colored in red. The 3′-UTR is underlined.

^
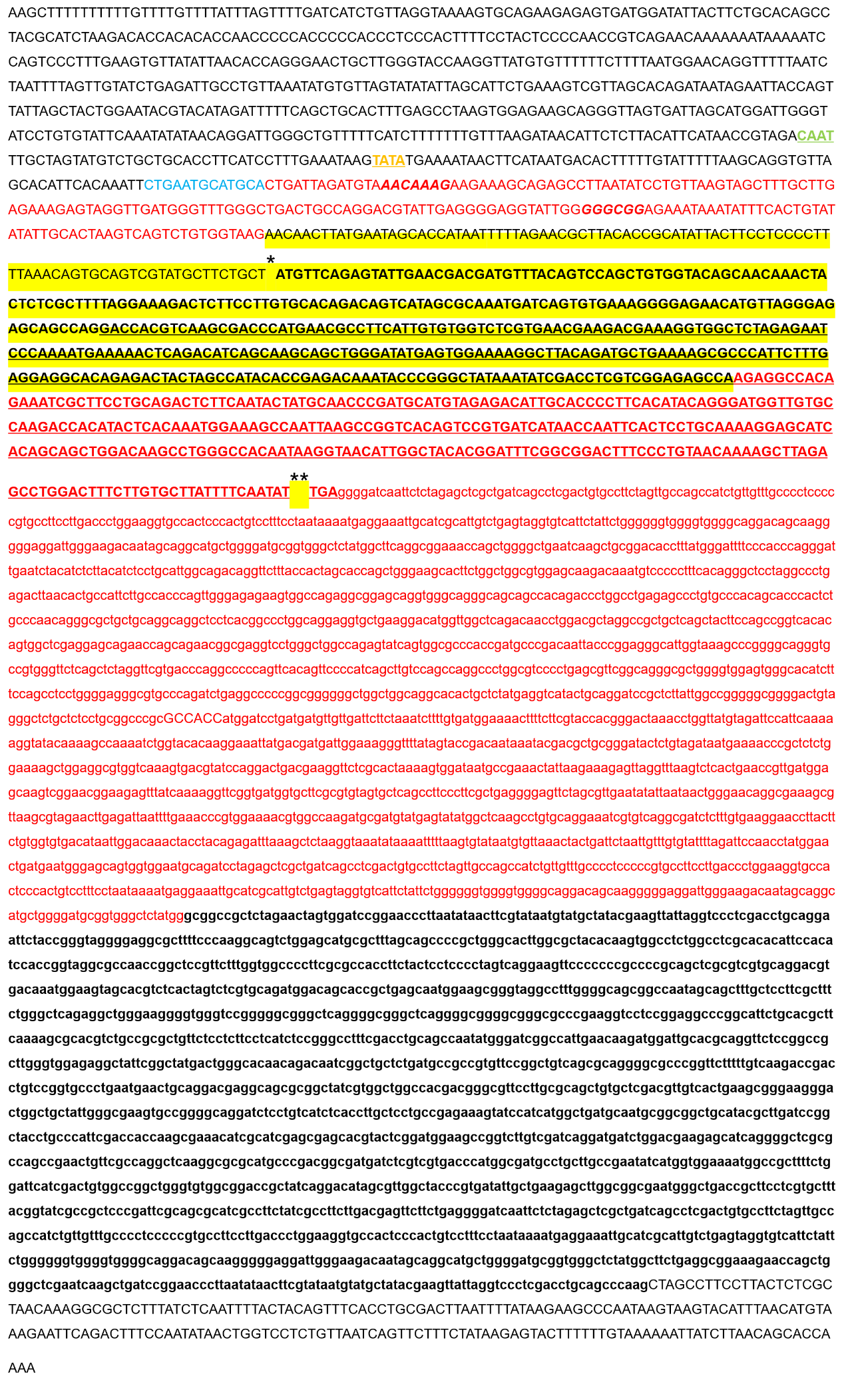
^

**Fig.S2** **Nucleotide sequence of the bovine with** **NHR targeting**

The uppercase sequence is the genome sequence of bovine *Sry*. Black text indicates the 651-bp 5′-UTR of the bovine *Sry* genomic fragment, which includes a TATA motif, underlined and colored in yellow, and a CAAT box, underlined and colored in green. The red text, which includes the SRY-binding motif (italicized) and the Spl-binding motif (italicized), indicates one of the fragments deleted after NHR gene targeting. The blue text indicates the bases inserted after NHR gene targeting. The text highlighted in bright yellow, which includes the 91-bp 5′-UTR and the 388-bp ORF (the ATG transcription start codon is indicated by *; the HOM box is bolded and underlined) of the bovine *Sry* genomic fragment, indicates the fragment that was inserted in reverse after NHR gene targeting. The red underlined text, which includes the 302-bp ORF (the TGA stop codon is indicated by **) of the bovine *Sry* genomic fragment, indicates the other fragment deleted after NHR gene targeting.

Lowercase text indicates the inserted gene in the pSRY-DTA vector. The red text including ployA-cPrm1-DTA is the sequence that was not inserted into the expected position. The bolded sequence including PGK-NEO is the sequence that was inserted into the expected position.


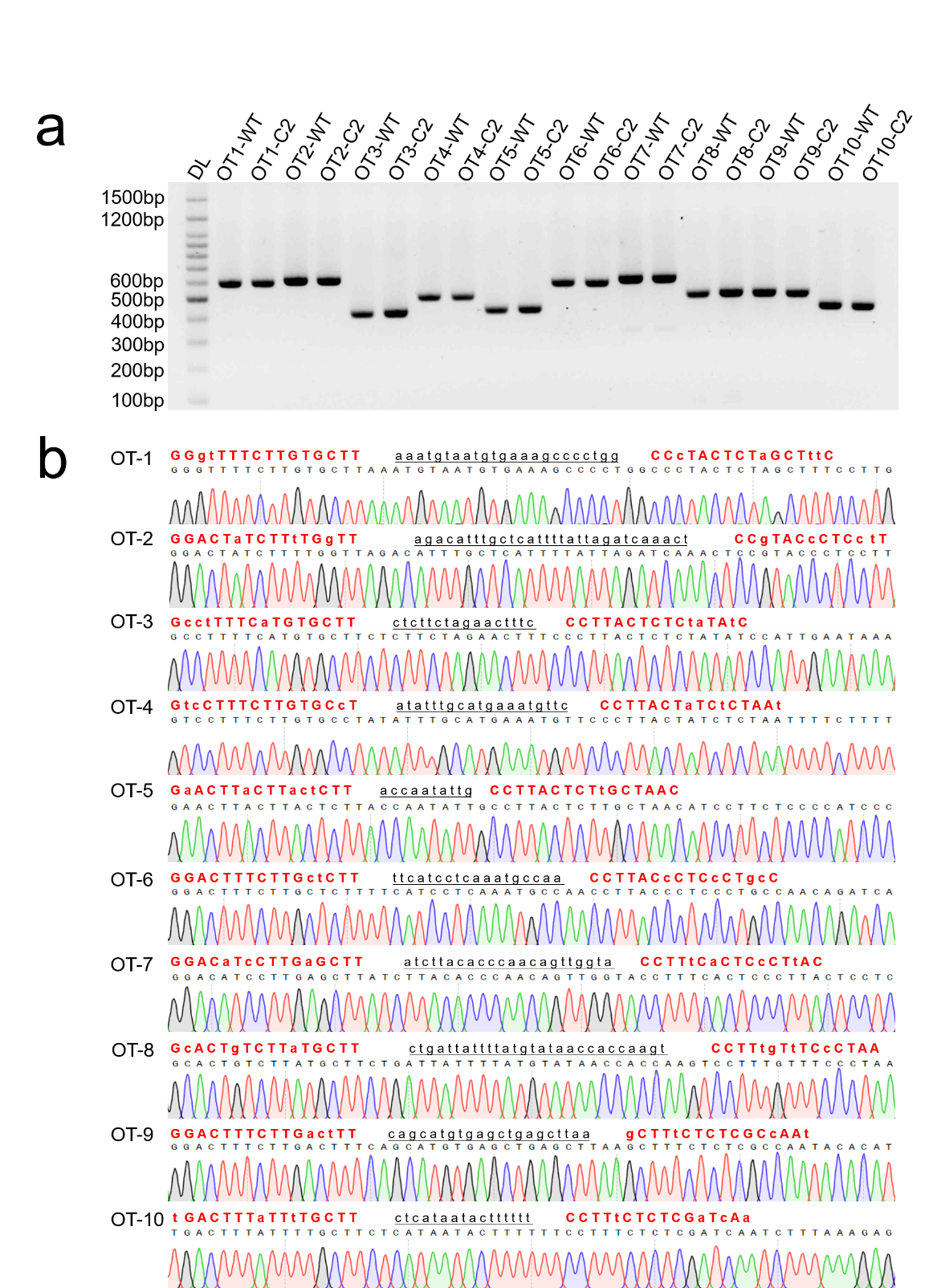


**Fig.S3 Off-target analysis of *Sry* TALEN pair 1 in sex-reversal heifer C2 (#160705)**

**(a)** T7EI cleavage analysis of the top 10 potential off-target sequences. OT-1- OT-10, top 10 potential off-target sites; DL, 100-bp DNA ladder; WT, genome from WT heifer; C2, genome from sex-reversal heifer C2 (#160705). **(b)** Chromatogram sequence analysis of the top 10 potential off-target sequences. The TALEN recognition sequences, including paired left and right recognition sequences (colored in red) and the spacer sequences (underlined) between left and right hits, are shown.


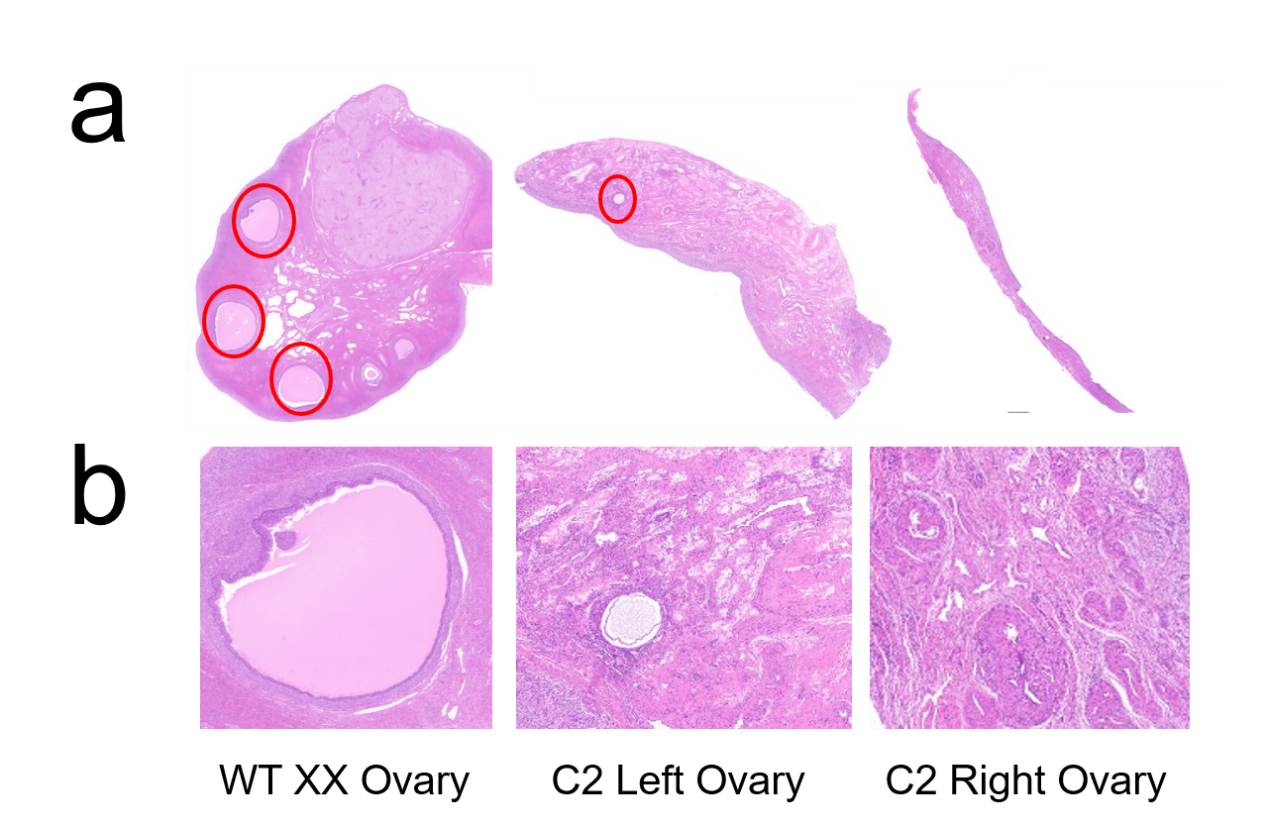


**Fig.S4 H****&E staining of the ovaries from a WT-XX cow and sex-reversal cow C2**

**(a)** Panoramic results of H&E staining. Red circles indicate follicles. **(b)** Follicular structure as shown by H&E staining. WT-XX, WT female control; C2, sex-reversal cow #160705.

**Table S1 TALEN recognition sequences and the amino acid sequence of** **the repeat variable di-residues (RVDs) in the corresponding TALENs**

| TALENs | TALEN recognition sequences (bold) and Amino acid sequence of the RVDs |
| --- | --- |
| *Sry*  Pair 1 | TGGACTTTCTTGTGCTT attttcaatattgactt CCTTACTCTCGCTAACA  Left: **GGACTTTCTTGTGCTT**  RVD: NN NN NI HD NG NG NG HD NG NG NN NG NN HD NG NG  Right: **GTTAGCGAGAGTAAGG**  RVD: NN NG NG NI NN HD NN NI NN NI NN NG NI NI NN NN |
| *Sry*  Pair 2 | TAGAGCCTGGACTTTCTTG tgcttattttcaatattg ACTTCCTTACTCTCGCTAA  Left: **AGAGCCTGGACTTTCTTG**  RVD: NI NN NI NN HD HD NG NN NN NI HD NG NG NG HD NG NG NN  Right: **TAGCGAGAGTAAGGAAGT**  RVD: NG NI NN HD NN NI NN NI NN NG NI NI NN NN NI NI NN NG |
| *Sry*  Pair 3 | TGGACTTTCTTGTGCTTAT tttcaatattgacttc CTTACTCTCGCTAACAAA  Left: **GGACTTTCTTGTGCTTAT**  RVD: NN NN NI HD NG NG NG HD NG NG NN NG NN HD NG NG NI NG  Right: **TTGTTAGCGAGAGTAAG**  RVD: NG NG NN NG NG NI NN HD NN NI NN NI NN NG NI NI NN |
| *ZFY*  Pair 1 | TGATGAGAATCAGAGGCC atggatgaagatga ATTTGAATTACAGCCACA  Left: **GATGAGAATCAGAGGCC**  RVD: NN NI NG NN NI NN NI NI NG HD NI NN NI NN NN HD HD  Right: **GTGGCTGTAATTCAAAT**  RVD: NN NG NN NN HD NG NN NG NI NI NG NG HD NI NI NI NG |
| *ZFY*  Pair 2 | TGGATGAAGATGAATTT gaattacagccacagga GCCAAACTCATGTTTTGA  Left: **GGATGAAGATGAATTT**  RVD: NN NN NI NG NN NI NI NN NI NG NN NI NI NG NG NG  Right: **CAAAACATGAGTTTGGC**  RVD: HD NI NI NI NI HD NI NG NN NI NN NG NG NG NN NN HD |
| *ZFY*  Pair 3 | TGAATTTGAATTACAGCC acaggagccaaactcat GTTTTGATGGAATAGGTA  Left: **GAATTTGAATTACAGCC**  RVD: NN NI NI NG NG NG NN NI NI NG NG NI HD NI NN HD HD  Right: **ACCTATTCCATCAAAAC**  RVD: NI HD HD NG NI NG NG HD HD NI NG HD NI NI NI NI HD |
| *DDX3Y*  Pair 1 | TCGCGCCTTTCTTCAGGC atgagtcatgaggc GGTGAAAAACGTGCAGGA  Left: **CGCGCCTTTCTTCAGGC**  RVD: HD NN HD NN HD HD NG NG NG HD NG NG HD NI NN NN HD  Right: **CCTGCACGTTTTTCACC**  RVD: HD HD NG NN HD NI HD NN NG NG NG NG NG HD NI HD HD |
| *DDX3Y*  Pair 2 | TCTTCAGGCATGAGTCAT gaggcggtgaaaaacgt GCAGGAGGTGGATCAGCA  Left: **CTTCAGGCATGAGTCAT**  RVD: HD NG NG HD NI NN NN HD NI NG NN NI NN NG HD NI NG  Right: **GCTGATCCACCTCCTGC**  RVD: NN HD NG NN NI NG HD HD NI HD HD NG HD HD NG NN HD |
| *DDX3Y*  Pair 3 | TGAGGCGGTGAAAAACG tgcaggaggtggat CAGCAGGTGAGTCAAGA  Left: **GAGGCGGTGAAAAACG**  RVD: NN NI NN NN HD NN NN NG NN NI NI NI NI NI HD NN  Right: **CTTGACTCACCTGCTG**  RVD: HD NG NG NN NI HD NG HD NI HD HD NG NN HD NG NN |
| *EIF2S3Y*  Pair 1 | TTTTGTTGCTCGTTCGG caagatggcgcggc GTGAGGAGTCATGTGTGA  Left: **TTTGTTGCTCGTTCGG**  RVD: NG NG NG NN NG NG NN HD NG HD NN NG NG HD NN NN  Right: **CACACATGACTCCTCAC**  RVD: HD NI HD NI HD NI NG NN NI HD NG HD HD NG HD NI HD |
| *EIF2S3Y*  Pair 2 | TCATGTGTGACCCTGGG tcagccacatcttt GTCGCCAGGATCTCTCCA  Left: **CATGTGTGACCCTGGG**  RVD: HD NI NG NN NG NN NG NN NI HD HD HD NG NN NN NN  Right: **GGAGAGATCCTGGCGAC**  RVD: NN NN NI NN NI NN NI NG HD HD NG NN NN HD NN NI HD |
| *EIF2S3Y*  Pair 3 | TGACCCTGGGTCAGCCAC atctttgtcgccag GATCTCTCCACACTGGTA  Left: **GACCCTGGGTCAGCCAC**  RVD: NN NI HD HD HD NG NN NN NN NG HD NI NN HD HD NI HD  Right: **ACCAGTGTGGAGAGATC**  RVD: NI HD HD NI NN NG NN NG NN NN NI NN NI NN NI NG HD |

**Table S2 Results of** **pSRY-EGFP cell screening**

| **Cell line** | **Screening method** | **Isolated**  **colonies** | | **Colonies analyzed by PCR** | | **Positive**  **cell colonies** | **HR Positivity**  **rate** | **Cells**  **frozen** |
| --- | --- | --- | --- | --- | --- | --- | --- | --- |
| mBFF1004 | G418 | 12 | 12 | | 2 | | 16.7% | #9、#11 |

**Table S3 Results of pSRY-DTA cell screening**

| **Cell line** | **Screening method** | **Isolated**  **colonies** | | **Colonies analyzed**  **by PCR** | | **Positive**  **cell colonies** | **HR Positivity**  **rate** | **Cell colonies selected for SCNT** |
| --- | --- | --- | --- | --- | --- | --- | --- | --- |
| mBFF1004 | G418 | 39 | 39 | | 1 | | 2.56% | #35 |

**Table S4 Microsatellite analysis of the sex-reversal cow**

| Site | Sample | | | |
| --- | --- | --- | --- | --- |
|  | MBFF1004 | | XY160705 | |
| TGLA227 | 100 | 102 | 100 | 102 |
| BM2113 | 123 | 125 | 123 | 125 |
| TGLA53 | 156 | 174 | 156 | 174 |
| ETH10 | 218 | 218 | 218 | 218 |
| SPS115 | 246 | 246 | 246 | 246 |
| TGLA126 | 117 | 117 | 117 | 117 |
| TGLA122 | 143 | 143 | 143 | 143 |
| INRA23 | 202 | 206 | 202 | 206 |
| ETH3 | 115 | 115 | 115 | 115 |
| ETH225 | 145 | 147 | 145 | 147 |
| BM1824 | 179 | 183 | 179 | 183 |

**Table S5 List of the top 10 potential off-target effects of *Sry* TALEN pair1**

| Potential off-target Coord | TALEN recognition sequences | Left match | Right match | SP |
| --- | --- | --- | --- | --- |
| chrY:42225190-42225239 | GGACTTTCTTGTGCTT **attttcaatattgactt** CCTTACTCTCGCTAACA | GGACTTTCTTGTGCTT | GTTAGCGAGAGTAAGG | 17 |
| chr1:110345446-110345501 | GGgtTTTCTTGTGCTT  **aaatgtaatgtgaaagcccctgg**  CCcTACTCTaGCTttC | GGgtTTTCTTGTGCTT | GaaAGCtAGAGTAgGG | 23 |
| chr5:55644314-55644375 | GGACTaTCTTtTGgTT  **agacatttgctcattttattagatcaaact** CCgTACcCTCctTAAC | GGACTaTCTTtTGgTT | GTTAagGAGgGTAcGG | 30 |
| chr6:13076996-13077043 | GcctTTTCaTGTGCTT  **ctcttctagaactttc**  CCTTACTCTCtaTAtC | GcctTTTCaTGTGCTT | GaTAtaGAGAGTAAGG | 16 |
| chr7:73726650-73726700 | GtcCTTTCTTGTGCcT **atatttgcatgaaatgttc**  CCTTACTaTCtCTAAt | GtcCTTTCTTGTGCcT | aTTAGaGAtAGTAAGG | 19 |
| chr7:112212246-112212287 | GaACTTaCTTactCTT  **accaatattg**  CCTTACTCTtGCTAAC | GaACTTaCTTactCTT | GTTAGCaAGAGTAAGG | 10 |
| chr10:90455160-90455209 | GGACTTTCTTGctCTT  **ttcatcctcaaatgccaa**  CCTTACcCTCcCTgcC | GGACTTTCTTGctCTT | GgcAGgGAGgGTAAGG | 18 |
| chr20:12890928-12890981 | GGACaTcCTTGaGCTT  **atcttacacccaacagttggta**  CCTTtCaCTCcCTtAC | GGACaTcCTTGaGCTT | GTaAGgGAGtGaAAGG | 22 |
| chr22:12466145-12466203 | GcACTgTCTTaTGCTT  **ctgattattttatgtataaccaccaagt**  CCTTtgTtTCcCTAA | GcACTgTCTTaTGCTT | GTTAGgGAaAcaAAGG | 28 |
| chr29:5398650-5398702 | GGACTTTCTTGactTT  **cagcatgtgagctgagcttaa**  gCTTtCTCTCGCcAAt | GGACTTTCTTGactTT | aTTgGCGAGAGaAAGc | 21 |
| chrX:79144197-79144244 | tGACTTTaTTtTGCTT  **ctcataatactttttt**  CCTTtCTCTCGaTcAa | tGACTTTaTTtTGCTT | tTgAtCGAGAGaAAGG | 16 |

“Potential off-targets Coord” indicates the positions of the TALEN pair1 hits in the cow genome assembly (chrom:start-end:strand). TALEN recognition sequences are the paired left and right recognition sequences and the spacer sequences between the left and right hits. Left match, the genomic sequence of the left hit (uppercase indicates a match with the consensus; lowercase indicates a mismatch). Right match, the genomic sequence of the right hit (uppercase indicates a match with the consensus; lowercase indicates a mismatch). SP, length of the spacer between the left and right hits.

**Table S6 Primers used in this study**

| Primer | Sequence |
| --- | --- |
| Sry-F | 5’ TGTGCCAAGACCACATACTC 3’ |
| Sry-R | 5’ AGAAAGACCAAAGAACAGACC 3’ |
| ZFY-F | 5’ TGACTCTTTGCGACCC 3’ |
| ZFY-R | 5’ TGAAAAGCAGGAATTCTATGG 3’ |
| DDX3Y-F | 5’ GTGTGATGGACGGGATTGGCT 3’ |
| DDX3Y-R | 5’ GCCATTGCTCCGGAATAAGG 3’ |
| EIF2S3Y-F | 5’ GCGACCTCACCATAAGA 3’ |
| EIF2S3Y-R | 5’ AAAAACTCCTTCCAGAACTGA 3’ |
| P1 | 5’ TGCTCGACGTTGTCACT 3’ |
| P2 | 5’ AATCAGACATTTATGCATTCC 3’ |
| P3 | 5’ TCCTCCTATTTTATTACATGA 3’ |
| P4 | 5’ CCTGGACGTAGCCTTC 3’ |
| P5 | 5’ TCTTACATTCATAACCGTAGA 3’ |
| P6 | 5’ CGCTTCAGTGACAACGTC 3’ |
| P7 | 5’ TCTTACATTCATAACCGTAGA 3’ |
| neo-F | 5’ GGCAGGTAGCCGGATCAAGCG 3’ |
| neo-R | 5’ CAAGATGGATTGCACGCAGGTTCTC 3’ |
| BSP-F | 5’ TTTACCTTAGAACAAACCGAGGCAC 3’ |
| BSP-R | 5’ TACGGAAAGGAAAGATGACCTGACC 3’ |
| BY-F | 5’ CTCAGCAAAGCACACCAGAC 3’ |
| BY-R | 5’ GAACTTTCAAGCAGCTGAGGC 3’ |
